# Rethinking Intravenous Catheter Size and Location for Computed Tomography Pulmonary Angiography

**DOI:** 10.5811/westjem.2018.11.40930

**Published:** 2019-02-06

**Authors:** Travis Marshall, Nae Meng Chen, Eric Nguyen, David E. Slattery, Tony Zitek

**Affiliations:** * University of Nevada, Las Vegas School of Medicine, Department of Emergency Medicine, Las Vegas, Nevada; † University Medical Center of Southern Nevada, Department of Emergency Medicine, Las Vegas, Nevada; ‡ Kendall Regional Medical Center, Department of Emergency Medicine, Miami, Florida

## Abstract

**Introduction:**

Computed tomography pulmonary angiography (CTPA) is the test of choice for diagnosis of pulmonary embolism (PE) in the emergency department (ED), but this test may be indeterminate for technical reasons such as inadequate contrast filling of the pulmonary arteries. Many hospitals have requirements for intravenous (IV) catheter size or location for CTPA studies to reduce the chances of inadequate filling, but there is a lack of clinical data to support these requirements. The objective of this study was to determine if a certain size or location of IV catheter used for contrast for CTPA is associated with an increased chance of suboptimal CTPA.

**Methods:**

This was a retrospective chart review of patients who underwent CTPA in the ED. A CTPA study was considered suboptimal if the radiology report indicated it was technically limited or inadequate to exclude a PE. The reason for the study being suboptimal, and the size and location of the IV catheter, were abstracted. We calculated the rate of inadequate contrast filling of the pulmonary vasculature and compared the rate for various IV catheter sizes and locations. In particular, we compared 20-gauge or larger IV catheters in the antecubital fossa or forearm to all other sizes and locations.

**Results:**

A total of 19.3% of the 1500 CTPA reports reviewed met our criteria as suboptimal, and 51.6% of those were due to inadequate filling. Patients with a 20-gauge IV catheter or larger placed in the antecubital fossa or forearm had inadequate filling 9.2% of the time compared to 13.2% for patients who had smaller IVs or IVs in other locations (difference: 4.0% [95% confidence interval, −1.7%–9.7%]). There were also no statistically significant differences in the rates of inadequate filling when data were further stratified by IV catheter location and size.

**Conclusion:**

We did not detect any statistically significant differences in the rate of inadequate contrast filling based on IV catheter locations or sizes. While small differences not detected in this study may exist, it seems prudent to proceed with CTPA in patients with difficult IV access who need emergent imaging even if they have a small or distally located IV.

## INTRODUCTION

Since the publication of the Prospective Investigation of Pulmonary Embolism Diagnosis II trial,^1^ computed tomographic pulmonary angiography (CTPA) has become the test of choice for diagnosis of pulmonary embolism (PE) in the emergency department (ED).^2^–^3^ The test characteristics of CTPA are reported to be quite good with sensitivity and specificity of 89% and 95%, respectively.^4^ While CTPA can be highly accurate when performed with proper technique, the reported sensitivity and specificity do not account for the times when CTPA is indeterminate because of technical factors such as motion artifact or inadequate filling of the pulmonary arteries.^5^

The American College of Radiology (ACR) recommends a 20-gauge or larger intravenous (IV) catheter in the antecubital fossa or forearm for CTPA.^6^ The ACR recommendations do not provide supporting references, and a literature search did not reveal published clinical data supporting these recommendations. Nonetheless, many hospitals have policies that follow them. While these policies are designed to improve the quality of CTPA, in patients with difficult IV access these policies may result in significant delays in diagnosis while ED staff attempt to establish an IV that follows hospital policies.

Thus, we performed a retrospective chart review to assess if a certain location or size of the IV catheter used for a contrast bolus for CTPA is associated with an increased chance of inadequate filling of the pulmonary vasculature. In particular, we sought to determine if the ACR recommendation that a 20-gauge or larger IV catheter in the antecubital fossa or forearm is associated with decreased rates of inadequate filling of the pulmonary vasculature on CTPA compared to other IV catheter sizes and locations.

## METHODS

### Study Design and Setting

This was a retrospective study performed at a single, large, urban, county hospital in Las Vegas, Nevada. The annual census of our adult ED is approximately 77,000. The CTPA studies from our adult ED are rapidly read 24 hours per day by a private group that currently employs 64 radiologists. The standard peripheral IV catheter used in our department is the 1.00-inch Becton Dickinson (BD) Insyte^TM^ Autoguard^TM^, which is available in sizes 16-gauge, 18-gauge, 20-gauge, and 22-gauge. In rare cases, a 2.5-inch, 18-gauge Introcan Safety® catheter is used for ultrasound-guided deep brachial IV lines or for placement in the internal jugular vein (“peripheral IJs”).^7^ This study received approval from our hospital’s institutional review board, which waived full review.

We identified adult patients who underwent CTPA in the ED to evaluate for PE. We were able to identify these patients because our radiology image-viewing software system allows us to search for patients based upon imaging study type and date. Patients were excluded if they had undergone CTPA for any reason other than to rule out PE. Of the patients meeting the inclusion criteria and not meeting the exclusion criteria, we extracted additional patient data including basic demographics, whether or not the CTPA was suboptimal, why the CTPA was suboptimal (if applicable), and the size and location of the IV line.

Two premedical student research assistants functioned as data abstractors. They were blinded from the study objectives, and they used standardized data collection forms to perform chart reviews. Each data abstractor was trained through the review of 20 sample charts with a physician investigator. They assessed each final, attending radiology impression to determine if the CTPA met our definition of “suboptimal.” We considered a CTPA suboptimal if the final radiology impression read any of the following: inadequate filling/suboptimal timing of the contrast bolus; motion artifact; or any case the radiology impression called the study technically limited or inadequate to exclude a PE. However, impressions stating inability to exclude subsegmental PE were not included as suboptimal, since subsegmental PEs may not need to be treated.^8^ Note that our definition of “suboptimal” is consistent with prior literature on this topic.^9^

**Population Health Research Capsule**

What do we already know about this issue?

*Many hospitals have requirements for intravenous (IV) catheter size or location for computed tomography pulmonary angiography (CTPA) studies to reduce the chances of a suboptimal study, but such requirements may result in delayed diagnosis*.

What was the research question?

Is the size or location of an IV catheter used for CTPA associated with an increased chance of inadequate contrast filling?

What was the major finding of the study?

*We did not find differences in the rate of inadequate contrast filling of CTPAs at various IV catheter locations or sizes*.

How does this improve population health?

*It may be prudent to proceed with CTPA in patients with difficult IV access who need emergent imaging even if they have a small or distally located IV*.

The data abstractors were periodically monitored, and a physician investigator audited 50 charts from each of the abstractors to assess for accuracy. Also, both abstractors reviewed a sample of 50 charts to assess the inter-rater reliability for the study.

All CTPA studies were performed on a 64-slice scanner (Siemens Medical Solutions USA Inc; Malvern, PA) with a standard CTPA protocol at the hospital where data were collected. This includes a localizer sequence through the carina followed by a timing bolus of 30-cubic centimeter (cc) contrast bolus of Optiray 350 (Ioversol 74%; Guerbet LLC; Princeton, New Jersey) to localize the pulmonary arteries until the maximum Hounsfield unit is measured. A 90-cc bolus is then injected at 4–5 cc/sec both preceded and followed by a 50-cc saline flush given through a power injector. Continuous 0.6 millimeter (mm) axial slices are taken from above the apices to below the costophrenic angles with an inspiratory hold. Our hospital’s protocol calls for 20-gauge IV access or greater at the antecubital fossa or forearm, but this can be overridden by the attending physician based on emergent indications. Pursuant to protocol, radiology technicians use a 10-cc normal saline flush to evaluate access prior to administration of contrast. Additional scanning parameters are as follows: 120 kilovolt peak (kVp), 2 × 2 mm reconstruction, pitch of 0.8–1.0, and coronal and sagittal multiplanar reformation of 3 × 3 mm.

### Outcomes and Data Analysis

As discussed below, we decided to review a sample of 1500 CTPA studies. After review, we calculated the percentage of all CTPA studies that were suboptimal, and determined the fraction of those suboptimal studies that were due to inadequate filling of the pulmonary vasculature.

The primary outcome for the study was meant to assess the ACR’s recommendations for IV size and location for CTPA. In particular, we aimed to measure the difference in the rate of inadequate filling of the pulmonary vasculature for 20-gauge or larger catheters in the antecubital fossa or forearm compared to the rate of inadequate filling for all other catheter size and location combinations.

Secondarily, the percentage of studies with inadequate filling of the pulmonary vasculature were stratified by IV catheter size and location. We compared the percentage of studies with inadequate filling when a 20-gauge or larger IV catheter was used to the percentage of studies with inadequate filling when smaller catheters were used. Also, the rate of inadequate filling was compared for IV catheters placed in the forearm or antecubital fossa to IV catheters placed at other locations.

Our initial choice of a sample size of 1500 was based on the size of a previously published study about suboptimal CTPAs^9^ and gestalt that this would be sufficiently large. Since no prior study has evaluated the relationship between IV size or location and suboptimal CTPAs, we initially did not have sufficient information to perform a formal power calculation. However, with the knowledge of the results of this study, we can provide a post-hoc power analysis as follows: for the primary outcome, assuming that patients would have an IV catheter meeting the ACR recommendations six times as often as not, we found that at least 132 patients would be required in the group not meeting the ACR recommendations to find a 10% difference in the rate of inadequate contrast filling of the pulmonary vasculature with a power of 0.8 and an alpha of 0.05.

Data were collected and analyzed via Microsoft Excel (Version 15, Microsoft, Redmond, Washington). We performed statistical analysis using “R” (version 3.5.2, R Foundation, Vienna, Austria). The proportions for each group were compared using Fisher’s exact test.

## RESULTS

A total of 1500 consecutive CTPA studies to assess for PE in our ED from June 2016 to March 2017 were identified and included for analysis. The patients upon which these studies were performed were 48.2% female. The median age was 55 years (interquartile range [IQR]: 42–65), and the median body mass index was 28 (IQR: 24–34). The patients were 56.8% Caucasian, 23.7% African American, 12.8% Hispanic, and 5.0% Asian.

Of the 1500 studies, 289 (19.3% [95% confidence interval {CI}, 17.3–21.4%]) met our criteria for suboptimal. Of the suboptimal studies, 51.6% (147/289) were due to an inadequate filling of the pulmonary vasculature. Table 1 shows the reasons why the CTPA studies were considered suboptimal.

Inter-rater reliability was determined based on the assessment of whether or not the CTPA was suboptimal from a sample of 50 charts, and Cohen’s kappa was 0.92 between the two student abstractors. A physician auditor abstracted 100 charts (50 done by each abstractor) to assess the inter-rater reliability between the physician and each of the abstractors. The two additional Cohen’s kappa values were calculated at 0.92 and 0.96.

Regarding the primary outcome, patients with a 20-gauge or larger IV catheter placed in the antecubital fossa or forearm (the ACR recommendations) had inadequate filling 9.2% of the time (81/883) compared to 13.2% (20/152) for patients who had smaller IVs or IVs in other locations. The difference of 4.0% (95% CI, −1.7%–9.7%) is not statistically significant.

When a patient had an IV catheter in the antecubital fossa or forearm, the rate of inadequate filling of the pulmonary vasculature was 9.3% (83/888), compared to 12.2% (18/147) in other IV locations. The difference between these groups was 2.9% (95% CI, −2.7%–8.5%), which is not statistically significant.

Only 13 patients had 22-gauge IV catheters for CTPA, but a comparison of the rate of inadequate filling for 22-gauge IV catheters (23.1 %) to larger catheters (9.7%) revealed a difference of 13.4% (95% CI, −9.6%–36.4%).

Unfortunately, the IV catheter location used for CTPA was not specified in the chart in 465 of the 1500 studies, and in 464 cases the size of the IV catheter used was not recorded. In an attempt to assess for bias that may have been introduced into the study from the missing IV data, we performed an additional analysis and found that the rate of inadequate contrast filling was nearly identical for patients who had an IV size recorded (9.9%) compared to those with missing data (9.7%). Similarly, the rates of inadequate contrast filling were nearly equal for patients with an IV location recorded (9.8%) and those without an IV location recorded (9.9%).

The chance of inadequate filling of the pulmonary vasculature was determined for each IV catheter size and location, as listed in Tables 2 and 3.

## DISCUSSION

To our knowledge, this is the largest study to evaluate the rate of suboptimal CTPA, and the only study to attempt to determine if a certain IV size or location is associated with an increased chance of inadequate filling of the pulmonary vasculature resulting in a suboptimal study. We found a fairly high rate of suboptimal CTPA, 19.3%. This number is substantially higher than the 4% found in a study by Bates et al. that used a similar definition of “suboptimal.”^9^ The chart review methods in that study were not as rigorous as ours, and we suspect the true suboptimal rate is higher than 4%.

While no other recent study has looked at the rate of suboptimal CTPA as it is defined in our study and the one by Bates et al, some other studies related to this issue are of note. For example, a study by Molaee et al. found that 7.9% of CTPA studies were of “unsatisfactory technique,” such that they could not be adequately interpreted.^10^ An older study from 2004 found that an artifact called “transient interruption of contrast” occurs in 37% of CTPA studies, limiting the radiologist’s ability to interpret the study.^11^ Additionally, another recent study related to this subject found that 9.5% of CTPA studies were “technically limited.”^12^ In the end, because of variations in local radiology practice styles, differences in technique for the execution of CTPA, and differences in equipment, the rate of suboptimal CTPA likely varies a bit from hospital to hospital.

Regardless of the exact rate of suboptimal CTPA, there is some consensus from previous studies^9^–^11^ and this study that inadequate filling of the pulmonary vasculature accounts for a large portion of the suboptimal CTPAs. Since suboptimal CTPAs could lead to unnecessary anticoagulation and additional testing, it is important to minimize the chances of a suboptimal CTPA.

Thus, it makes sense to put forth recommendations about the IV size and location if these recommendations will reduce the frequency of suboptimal CTPA. While our study does show trends toward reductions in the rate of inadequate filling of the pulmonary vasculature when larger IVs in the antecubital fossa or forearm are used, the difference in the rates of inadequate filling for various IV sizes and locations appears to be small. Moreover, even patients with ideally located, 18-gauge IV catheters have inadequate filling of the pulmonary vasculature about one in 10 times, suggesting that factors other than the IV size and location affect the quality of the contrast bolus.

While our sample size for patients with 22-gauge IVs was very small, it is notable that 10 of 13 patients with these small IVs had CTPAs with completely adequate filling of the pulmonary vasculature. Interestingly, the packaging for a 22-gauge BD Insyte^TM^ Autoguard^TM^ catheter lists the maximum flow rate as 35 mL per minute, which should not allow for the standard rapid contrast bolus of 4–5 cc/second for a CTPA. However, through direct communication with BD Medical, we confirmed that the maximum listed flow rate is the gravity flow rate, and they claim that the BD Insyte^TM^ Autoguard^TM^ can be safely used for power injection as long as the pressure is limited to 300 pounds per square inch. Moreover, prior data suggests that 22-gauge peripheral IV catheters can tolerate the high flow rates from power injection without risking material damage.^13^

Thus, 22-gauge IV catheters can likely be safely and adequately used for CTPA, and some data suggest that even intraosseous lines can be used for CT angiography. A tibial intraosseous line has been reported to have been used for successful administration of contrast for a CTPA study, with excellent opacification of the pulmonary arteries,^14^ and a humeral intraosseous line been successfully used for a CT angiogram of the chest and abdomen.^15^

With regard to the use of unusual IV locations for CTPAs, the data we found for neck IVs are small but interesting. In these cases, neck IVs refer to external jugular vein IVs and peripheral IVs, and IVs in this location had very low rates of inadequate contrast filling of the pulmonary vasculature. Perhaps this is due to the nearly direct route from the external or internal jugular vein to the superior vena cava. A potential downside to the use of neck lines for CTPAs is that contrast extravasation may be more dangerous in the neck than in other locations of the body, but this was not assessed in our study.

The hospital where this study was performed allows the physician to proceed with CTPA even if the IV is smaller than recommended or not in the antecubital fossa or forearm in emergent situations. Based upon the results of our study, this appears to be a reasonable and important exception to the ACR recommendations for IV size and location. We hope that CTPA will not be delayed in an unstable patient with difficult IV access just because the IV size or location does not meet the recommendations. If the line is tested before contrast injection with a saline flush, there is no resistance, and there are no other easily obtainable IV access sites, it is reasonable to proceed with CTPA regardless of the IV size or location.

## LIMITATIONS

Our study had several limitations. First, this was a retrospective study, which raises the possibility of confounders and unrecognized bias. Second, this was a single-center study with a single radiology group, which limits the external validity of the study. Additionally, while the study was adequately powered for the primary outcome, our sample sizes for some of the secondary outcomes were small. Thus, the data trends we observed may have become statistically significant with larger sample sizes.

Next, there was a fair amount of missing data in our IV size and location in analysis. However, our analysis of the missing data found that the rates of inadequate filling of the pulmonary vasculature were nearly identical for those patients with missing data compared to those with complete data for IV size and location. This suggests that the missing data would have been unlikely to have made a dramatic change to our results. Another issue related to missing data regards IV catheter length. Although the IV catheter length could be related to the rate of inadequate filling, the IV catheter length is generally not recorded in our electronic health record system. Therefore, we could not do a formal analysis of IV catheter length. However, central lines (which are, of course, longer than typical peripheral IV catheters) were separated out from the peripheral IVs in our analysis. Also, we know that the only available long IV catheter in our department is a 20-gauge, and this catheter is only used for upper arm and neck IV-line placement. With this information, the maximum possible number of long IV catheters was 23, making up only 2% of the total sample of 20-gauge or larger IV size group. Thus, variable IV catheter length was not much of a factor in our study.

## CONCLUSION

Suboptimal CTPA reports occurred nearly 20% of the time in this study, more than half of which were due to inadequate filling of the pulmonary vasculature. While larger IVs in the antecubital fossa or forearm may slightly reduce the rate of inadequate contrast filling of the pulmonary arteries, we were unable to find any statistically significant differences in the rates of inadequate filling based on IV size or location. In emergent situations, the physician should proceed with CTPA even if an IV line meeting the ACR recommendations cannot be established.

## Figures and Tables

**Table 1:** Reasons for suboptimal CTPA studies.

Reason for suboptimal study	Percent of suboptimal studies
Motion artifact	54.3% (157/289)
Inadequate filling	51.6% (147/289)
Other	2.1% (6/289)
Multifactorial	11.4% (33/289)

*CTPA*, computed tomography pulmonary angiography.

**Table 2:** Intravenous (IV) catheter location and rate of inadequate pulmonary vasculature filling.

IV location	Total # (%)	Rate of inadequate filling
Antecubital	669 (64.6%)	62/669 (9.3%)
Forearm	219 (21.2%)	21/219 (9.6%)
Neck	38 (3.7%)	3/38 (7.9%)
Hand	38 (3.7%)	7/38 (18.4%)
Wrist	37 (3.6%)	4/37 (10.8%)
Upper arm	19 (1.8%)	2/19 (10.5%)
Central line	12 (1.2%)	2/12 (16.7%)
Leg	3 (0.3%)	0/3 (0%)

**Table 3:** Intravenous (IV) catheter size and rate of inadequate pulmonary vasculature filling.

IV size	Total # (%)	Rate of inadequate filling
16-gauge	3 (0.3%)	0/3 (0%)
18-gauge	316 (30.5%)	33/316 (10.4%)
20-gauge	704 (68.0%)	66/704 (9.4%)
22-gauge	13 (1.3%)	3/13 (23.1%)
